# Prevalence, Incidence, and Clinical Characteristics of Thyroid Eye Disease in Japan

**DOI:** 10.1210/jendso/bvad148

**Published:** 2023-11-27

**Authors:** Natsuko Watanabe, Ai Kozaki, Kosuke Inoue, Hiroto Narimatsu, Masahiko Shinohara, Michael Goddard LoPresti

**Affiliations:** Department of Internal Medicine, Ito Hospital, Shibuya-ku, Tokyo, 150-8308 Japan; Olympia Eye Hospital, Shibuya-ku, Tokyo, 150-0001 Japan; Department of Social Epidemiology, Graduate School of Medicine, Kyoto University, Sakyo-ku, Kyoto, 606-8501 Japan; Cancer Prevention & Cancer Control Division, Kanagawa Cancer Center Research Institute, Yokohama, Kanagawa, 241-8515 Japan; Real World Evidence Department, INTAGE Healthcare Inc., Chiyoda-ku, Tokyo, 101-0062 Japan; Value and Access Department, INTAGE Healthcare Inc., Chiyoda-ku, Tokyo, 101-0062 Japan

**Keywords:** thyroid eye disease, incidence, prevalence, epidemiology, treatment, claims database

## Abstract

**Background:**

Although thyroid eye disease (TED) can impact social and psychological well-being, the epidemiological evidence of TED is lacking in Japan.

**Methods:**

Nationwide claims databases provided by JMDC Inc. and Medical Data Vision Co., Ltd. and national population statistics are used. Three TED definitions ranging from a strict definition only including a TED diagnosis to a broad definition including a TED diagnosis and considering ocular symptoms are considered. The proportion of patients by severity and disease activity are estimated based on definitions that would allow identification of those patients within the claims data.

**Results:**

The incidence rate per 100 000 person-years ranged from 7.3 to 11.1 for the strict and broad TED definitions, respectively. For fiscal year 2020 (April 2020 to March 2021) the prevalence rate ranged between 24.65 (strict TED) and 37.58 (broad TED) per 100 000 persons. These correspond to 25 383 and 38 697 patients for the strict and broad TED definitions, respectively. Regardless of the definition used, a predominance of female patients was observed, and the highest burden of the disease was seen in the age group of 35 to 59. Mild and inactive forms of TED were predominant (about 85% and 74%, respectively).

**Conclusion:**

The incidence and prevalence of TED in Japan were 7.3 to 11.1 per 100 000 person-years and 24.65 to 37.58 per 100 000 persons, respectively. The robust results of this database study add valuable real-world evidence on the incidence and prevalence of TED in Japan.

Thyroid eye disease (TED), also called Graves’ orbitopathy or ophthalmopathy and thyroid associated ophthalmopathy, is an autoimmune disorder of the orbit that has been reported to be associated with Graves’ disease (GD) and chronic thyroiditis [1, 2]. The annual incidence of TED in the United States’ general population has been reported to be 16 per 100 000 person years (py) in women and 3 per 100 000 py in men [3]. The overall incidence of TED in Sweden has been estimated at 4.2 per 100 000 persons with a 3.9:1 female-to-male (F/M) ratio and in Denmark at 5.0 per 100 000 py, with 8.0 per 100 000 py for women and 1.9 per 100 000 py for men [4, 5]. Moreover, the approximate prevalence of TED has been reported to be 250 per 100 000 persons in the United States, 90 to 155 per 100 000 persons in Europe, and 100 to 300 per 100 000 persons in Asia [1, 3, 6].

The clinical presentation of TED has been shown to vary by age and sex [1, 6, 7]. TED has been shown to be more frequent in women than in men, with the F/M ranging from 2.1 to 4.2 across different studies. Sex also affects severity of the disease with the F/M ratio progressively decreasing with increasing severity and advanced age [3, 8]. In terms of severity, TED is often mild and self-limiting but may become sight-threatening in 3% to 5% of cases [2]. TED is also characterized by 2 phases: an active inflammatory (or acute) phase and an inactive noninflammatory (or chronic) phase. In TED's active inflammatory phase, progressive inflammation, swelling, and tissue changes occur. The active phase is then followed by the inactive/chronic noninflammatory phase where the disease progression stops. These inactive and active phases are often measured by a clinical activity score (CAS) ranging between 0 and 7, with the active phase being defined as a CAS ≥3 [7, 9].

Given that TED can impact patients’ social and psychological well-being, it is critical to understand who is more at risk to develop TED and its manifestation in terms of disease severity. Available data on the prevalence and incidence of TED are mainly from other countries, and epidemiological data on TED in Japan is lacking. Moreover, information on the characteristics of TED patients in Japan primarily come from studies that involve a single treatment center only, despite the potential for geographic variations associated with TED. Using nationwide data from JMDC Inc. and Medical Data Vision Co., Ltd. (MDV) for the period April 2012 to March 2021 [based on Japanese fiscal years (FY)], we calculated the incidence and national prevalence estimates of TED in Japan. We also clarified patients’ sociodemographic and clinical characteristics.

## Materials and Methods

### Study Design and Data Source

A cross-sectional analysis using nationwide insurance claims data from JMDC Inc. was conducted for the baseline analysis of incidence, prevalence, and patient and clinical characteristics. Moreover, hospital insurance claims data from MDV and national population statistics were used to estimate national prevalence of the disease [10]. The current database study includes data from April 2012 to March 2021 (based on the Japanese fiscal year of April to March) for those aged 18 or older.

The JMDC database is a commercially available health insurance claims database that includes enrolees of employer-managed health insurance with accumulated claims (inpatient, outpatient, prescriptions, procedures, etc.) and health check-up data from multiple health insurance societies since 2005. Employer-managed health insurance is one of the major types of health insurance in Japan whereby employees and their dependents are eligible for health insurance that is managed by their employer. This allows them access to healthcare reimbursed under the national health insurance system. The JMDC database includes a cumulative population of approximately 14 million unique enrollees [11]. Because JMDC data also includes full information on the number, age, and sex of all insurance enrollees for the insurance programs it covers, the data enables robust epidemiological examination for the general population, including healthy people, and also for tracking of patients, even if they transfer to other hospitals or undergo medical examinations at multiple facilities. For that reason, the JMDC database has been used to estimate the prevalence and incidence of numerous conditions for Japan [12, 13, 14].

However, due to the fact that the JMDC database is comprised of enrollees from employer-managed health insurance programs, it includes only a limited proportion of persons aged 60 or older. To account for this issue, estimates of the age and sex distribution of adult patients with TED obtained from the MDV hospital-based claims database were used to adjust the estimate of the prevalence of patients aged 60 or older obtained from the JMDC database. This was done to better reflect the age distribution of the population. The MDV database is a Japanese hospital-based claims database that includes approximately 40 million unique patients [11]. National-level population statistics for Japan available from the Statistics Bureau, Ministry of Internal Affairs and Communication are used to calculate national estimates of the prevalence of TED among adults aged 20 or older in Japan. The study was conducted in accordance with the Declaration of Helsinki. Permission for use of the data was obtained from health insurance societies and medical institutions by the data providers after undergoing anonymous processing. Based on the Ethical Guidelines for Medical and Health Research Involving Human Subjects issued by the Ministry of Health, Labor, and Welfare in Japan, studies involving the use of processed and anonymized claims data are exempt from receipt of informed consent and institutional board review [15].

### Patient Population

Each diagnosis and claim in Japan is associated with a specific disease name that is indicated by the treating physician when filing a claim, and “thyroid eye disease” is an established disease name available for the purpose of filing a claim in Japan. However, there are known to be alternative disease names used for TED such as “Graves’ ophthalmopathy” and “thyrotoxic ocular protrusion.” In fact, historically “thyrotoxic ocular protrusion” or “Graves’ ophthalmopathy” was more commonly used in the literature for Japan published prior to 1990, and those diagnosis names may still be used by some physicians in Japan [1, 16, 17]. While these disease names may be viewed as synonymous in clinical practice, they are included as separate disease names in claims databases in Japan.

TED disease may also be diagnosed based on the presence of other autoimmune or thyroid diseases, together with ocular symptoms such as diplopia and proptosis that may have developed before a clear TED diagnosis is made. As such, 3 definitions of TED are considered in this study (Table 1). Briefly, definition 1 is a “strict” definition that consists of cases with the disease name “thyroid eye disease” indicated for the claim. The second “expanded” definition (definition 2) includes the disease names “thyroid eye disease,” “Graves’ ophthalmopathy,” or “thyrotoxic ocular protrusion” as qualifying diagnosis names. The third “broad” definition (definition 3) includes any disease covered in the “expanded” definition or a combination of another specific autoimmune or thyroid disease and 1 or more relevant ocular symptoms. All 3 definitions exclude suspected cases whereby physicians may have indicated a temporary diagnosis for diagnostic purposes. In the Supplemental Material, Supplemental Figure 1 shows the patient flow for the analysis [18].

**Table 1.** Definitions used for TED

Definition 1 (“strict”)	Patients with a disease name of “thyroid eye disease,” excluding suspected cases
Definition 2 (“expanded”)	Patients, excluding suspected cases, with 1 or more of the following injury or disease names: “thyroid eye disease,” “Graves’ ophthalmopathy,” or “thyrotoxic ocular protrusion”
Definition 3 (“broad”)	Patients, excluding suspected cases, with 1 or more of the following injury or disease names: “thyroid eye disease,” “Graves” ophthalmopathy,” or “thyrotoxic ocular protrusion” – OR – Patients with any of the following autoimmune and/or thyroid diseases AND 1 or more of the following ocular symptoms, excluding suspected cases: [Diseases] hyperthyroidism, hypothyroidism, chronic thyroiditis, Graves’ disease [Symptoms] diplopia, proptosis, optic neuritis, optic neuropathy

### Outcome Measures

The primary outcome measures for this study are the incidence and prevalence of TED among adults (defined as ≥ 18 years old) in Japan. The calculation of incidence was based on insured persons aged 18 or older (age at registration of insurance) who were continuously registered in the insurance registry since April 2012 and who had not developed TED up to March 2013. The incidence rates per 100 000 py were assessed for the entire 8-year period between April 2013 and March 2021.

The prevalence of TED was calculated for each FY between 2012 and 2020. Prevalent cases were those having at least 1 administrative claim for the relevant definition for the given fiscal year (FY 2012 to FY 2020). The national prevalence was then estimated based on national population statistics for Japan by sex and by using 5-year age intervals. As the Japanese national population statistics and vital statistics do not provide data for the 18 to 19 age group, only the 20 years or older age categories were considered for the national prevalence analysis. An explanation of the process used to adjust the prevalence and to calculate the national prevalence is provided in Supplemental Material, Method section [18].

The secondary measures of interest for this study included baseline demographics and clinical characteristics of patients aged 18 years or older at study index date, defined as the first physician diagnosis of TED. For this analysis the time of initial diagnosis based on the relevant definition was used as the index date. Patients who developed TED less than 1 year after enrolling were excluded because they may have been treated prior to enrolling. In addition to the age and sex of TED patients, the study aimed to describe disease severity, disease activity, common comorbidities and concomitant treatments prescribed, and procedures undergone during the study period. With the exception of age and sex, treatment characteristics were considered for a 2-year period following the index date, with a 1-year pre-index date wash-out period prior to a TED diagnosis. Patients aged 18 years or older (age at onset of TED) who were registered in the insurance registry from April 2012 or later and had developed TED were included.

Mild patients were defined as patients who did not receive related treatment (ie, oral or intravenous (IV) glucocorticoids and/or an ophthalmic surgery/procedure) during a 2-year period from initial diagnosis following a 1-year pre-index period prior to a TED diagnosis. Patients who only received subcutaneous or subtenon injections of triamcinolone acetonide and not oral or IV glucocorticoids and/or an ophthalmic surgery/procedure were included as mild patients based on the treatment guidelines for TED in Japan [19]. Local administration of triamcinolone acetonide is recommended in the guidelines in Japan to reduce symptoms related to inflammation, such as eye lid swelling or retraction, and is considered a milder form of treatment.

Moderate to severe patients were those who underwent oral or IV glucocorticoids and/or eye surgery (eg, orbital decompression, strabismus surgery, ocular muscle transposition, and/or lagophthalmos correction) and/or orbital radiation therapy during a 2-year period from initial diagnosis following a 1-year pre-index period prior to a TED diagnosis. Immunosuppressive agents, such as rituximab or tocilizumab, may also be used for those who do not respond to currently approved medications for TED in Japan. However, those medications are used off-label, and an accurate analysis through insurance claims data is not possible. Lastly, patients who were newly diagnosed with optic neuropathy or optic neuritis during the 2-year period following the 1-year pre-index period were defined as the most severe.

Historically, disease activity has been measured by a CAS ranging between 0 to 7. However, definitions used for severity and disease activity for this study do not align precisely with the diagnostic and treatment guidelines used for TED in Japan, due to the inherent limitations of insurance claims data, namely due to the fact that insurance claims data do not include CAS scores. Patients were therefore defined as active when 1 or more of the following prescriptions or procedures were implemented once or more during a 2-year period, following initial diagnosis following and a 1-year pre-index period prior to a TED diagnosis: methylprednisolone (injection), prednisone (oral medication), triamcinolone acetonide (injection, etc.), orbital radiotherapy, and/or eye surgery (eg, orbital decompression, strabismus surgery, ocular muscle transposition, lagophthalmos correction). International Classification of Diseases-10 codes used to identify comorbidities, Anatomical Therapeutic Chemical codes used to identify treatment, and a description of criteria for severity and disease activity are presented in Supplement Material, Supplemental Table S2 and Table S3 [18].

### Statistical Analysis

In all analyses performed, continuous variables are presented as mean values with standard deviations, and categorical variables are presented as absolute and/or relative frequencies. Statistical analysis was conducted using SAS® Enterprise Guide and System Release 9.4. Average annual percent change was assessed using Joinpoint Regression Program, Version 4.9.1.0—April 2022, Statistical Methodology and Applications Branch, Surveillance Research Program, National Cancer Institute.

## Results

### Patient Baseline Characteristics

Table 2 presents the baseline characteristics of patients identified using the JMDC Inc. insurance claims database for the most recent FY (FY 2020; April 2020 to March 2021). A total of 2367, 3234, and 3613 cases of TED were identified using the strict TED definition (definition 1), expanded definition (definition 2), and broad definition (definition 3), respectively. Across the 3 different definitions, a female predominance was observed. Mean age of patients at diagnosis was in the mid-40s—with about 60% to 69% of patients being in the age group 35 to 59 years.

**Table 2.** Patient baseline demographics based on the JMDC database for fiscal year 2020

	Definition 1 (Strict)	Definition 2 (Expanded)	Definition 3 (Broad)
Total	2367	3234	3613
Sex %			
Male	26.7	26.4	27.0
Female	73.3	73.6	73.0
Female to male ratio	3.2:1	3.3:1	3.2:1
Age groups %			
18-20	.1	.1	.1
20-24	1.7	1.7	1.7
25-29	3.5	3.3	3.1
30-34	5.1	5.2	4.9
35-39	7.1	7.1	6.7
40-44	9.3	10.4	10.0
45-49	15.0	15.2	14.7
50-54	13.8	14.2	14.3
55-59	12.0	11.8	11.8
60-64	7.1	6.8	6.8
65-69	6.9	6.5	6.6
70-74	7.3	6.8	7.0
75-79	5.2	5.0	5.4
80-84	3.3	3.4	3.9
≥85	2.5	2.5	2.9
Mean age at diagnosis	44.7	44.6	45.2
Median age (range)	45.5 (19-73)	45.0 (19-74)	45.5 (19-74)

### Incidence

Table 3 presents the estimated incidence per 100 000 py for TED among adults in Japan over the 8-year study observation period from FY 2013 to FY 2020 by TED definition and demographic characteristics. The overall estimated incidence per 100 000 py was 7.3 for the strict definition, 9.6 for the expanded definition, and 11.0 for the broad definition. As expected, disease incidence was higher in females as compared to incidence in males. The peak incidence across the 3 definitions was observed in the 55 to 59 age group (9.6, 11.9, and 15.1 per 100 000 py).

**Table 3.** Incidence rate per 100 000 person years of TED among adults from fiscal year 2013 to fiscal year 2020, by patient definition and demographics

	Definition 1 (Strict)	Definition 2 (Expanded)	Definition 3 (Broad)
Total person-years	6 579 832	6 579 508	6 579 333
Total incidence patients	479	629	725
Overall incidence	7.3	9.6	11.0
Sex			
Male	3.6	5.1	6.1
Female	13.0	16.7	18.7
Age groups			
18-19	3.8	8.3	8.3
20-24	3.5	5.0	5.0
25-29	6.6	8.0	8.5
30-34	6.7	9.4	10.7
35-39	7.3	9.8	11.0
40-44	8.2	11.5	12.6
45-49	9.2	11.1	12.5
50-54	7.3	9.4	12.0
55-59	9.6	11.9	15.1
60-64	6.1	6.1	10.4
≥65	3.1	4.1	10.3

Abbreviations: TED, thyroid eye disease.

### Prevalence

Table 4 shows the estimated national prevalence by fiscal year. Over the 9-year period from 2012 to 2020, there has been an increase in the prevalence of TED in Japan across the 3 definitions. The highest average annual percentage change was 5.2% (95% confidence interval [3.5; 6.9]). For FY 2020, the national prevalence rate of TED in Japan among adults aged 20 years or older was 24.65, 33.91, and 37.58 per 100 000 persons for the strict, expanded, and broad definitions, respectively. The corresponding counts of prevalent cases were 25 383, 34 913, and 38 697, respectively.

**Table 4.** Prevalence of TED among adults from fiscal year 2012 to fiscal year 2020, by patient definition

	National prevalence rate (persons per 100 000 persons aged 20 or older)			National prevalence (adults aged 20 or older)		
	Definition 1 (Strict)	Definition 2 (Expanded)	Definition 3 (Broad)	Definition 1 (Strict)	Definition 2 (Expanded)	Definition 3 (Broad)
FY 2012	17.00	28.93	32.14	17 610	29 960	33 289
FY 2013	18.66	32.46	36.13	19 305	33 588	37 384
FY 2014	20.93	35.62	39.25	21 642	36 837	40 589
FY 2015	22.84	36.17	40.32	23 654	37 460	41 759
FY 2016	24.03	35.26	39.37	24 862	36 477	40 731
FY 2017	23.73	34.27	38.41	24 527	35 410	39 696
FY 2018	24.42	34.96	39.08	25 199	36 078	40 330
FY 2019	25.83	35.90	39.63	26 613	36 988	40 823
FY 2020	24.65	33.91	37.58	25 383	34 913	38 697
Average annual percent change	5.2% (95% CI [3.5; 6.9])	2.2% (95% CI [.3; 4.1])	2.2% (95% CI [.6; 3.8])	—	—	—

Abbreviations: CI, confidence interval; FY, fiscal year; TED, thyroid eye disease.

Table 5 presents the national prevalence of TED among adults by age and sex for FY 2020. Similar to incidence, women had higher prevalence rates than males and the highest prevalence rate was observed among those aged 35 to 59 years old for all 3 definitions.

**Table 5.** Prevalence rate of TED among adults by age and sex for fiscal year 2020, by patient definition and demographics

		National prevalence rate (persons per 100 000 persons aged 20 or older)			National prevalence (adults aged 20 or older)		
		Definition 1 (Strict)	Definition 2 (Expanded)	Definition 3 (Broad)	Definition 1 (Strict)	Definition 2 (Expanded)	Definition 3 (Broad)
Total		24.65	33.91	37.58	25 383	34 913	38 697
Sex	Male	12.19	16.50	18.71	6038	8175	9269
	Female	36.20	50.04	55.07	19 345	26 738	29 428
Age	20-39	20.63	28.07	29.80	5253	7147	7588
	20-24	12.14	16.68	18.80	718	987	1112
	25-29	17.78	22.73	23.68	1058	1353	1409
	30-34	23.21	32.51	34.20	1477	2069	2176
	35-39	27.67	37.88	39.99	2000	2738	2891
	40-59	42.75	60.07	66.15	14 670	20 616	22 703
	40-44	32.32	49.24	53.34	2670	4067	4406
	45-49	44.19	61.13	66.09	4277	5917	6397
	50-54	45.35	63.70	71.51	3886	5459	6128
	55-59	49.13	66.24	73.91	3837	5173	5772
	≥60	13.38	17.52	20.60	5460	7150	8406
	60-64	16.21	21.26	23.96	1192	1563	1762
	≥65	12.76	16.70	19.86	4268	5587	6644

Abbreviations: TED, thyroid eye disease.

### Patient Clinical Characteristics

#### Disease severity and activity

Table 6 shows the clinical characteristics associated with TED for FY 2020 based on a 2-year period of treatment following initial diagnosis and a 1-year pre-index period prior to a TED diagnosis. Across the 3 definitions of TED, about 84% of patients had a mild form of the disease while about 2.5% to 3.0% of patients had a more severe form of the condition. With regard to disease activity, 72.8%, 74.1%, and 74.4% of patients for the strict, expanded, and broad definitions, respectively, had an inactive/chronic noninflammatory form of the disease.

**Table 6.** Clinical characteristics of TED patients, by patient definition

	Definition 1 (Strict)	Definition 2 (Expanded)	Definition 3 (Broad)
Total, n	1852	2275	2613
Severity, n (%)			
Mild	1553 (83.9)	1925 (84.6)	2206 (84.4)
Moderate-severe	243 (13.1)	292 (12.8)	325 (12.4)
Most severe	56 (3.0)	58 (2.5)	82 (3.1)
Disease activity, n (%)			
Inactive/chronic noninflammatory	1349 (72.8)	1686 (74.1)	1945 (74.4)
Active/acute inflammatory	503 (27.2)	589 (25.9)	668 (25.6)
Comorbidities/symptoms, n (%)			
Graves’ disease	1312 (70.8)	1634 (71.8)	1713 (65.6)
Hyperthyroidism	686 (37.0)	828 (36.4)	922 (35.3)
Chronic thyroiditis	175 (9.4)	227 (10.0)	318 (12.2)
Hypothyroidism	132 (7.1)	169 (7.4)	305 (11.7)
Hypertension	460 (24.8)	559 (24.6)	653(25.0)
Dyslipidemia	367 (19.8)	442 (19.4)	556 (21.3)
Diabetes	252 (13.6)	307 (13.5)	426 (16.5)
Treatment, n (%)			
Thyroid preparations	1233 (66.6)	1506 (66.2)	1583 (60.6)
Eye preparations	878 (47.4)	1034 (45.5)	1142 (43.7)
Lubricants	668 (36.1)	791 (34.8)	859 (32.9)
Oral or injectable analgesics	366 (19.8)	459 (20.2)	552 (21.1)
Oral or injectable anti-inflammatories	330 (17.8)	421 (18.5)	499 (19.1)
Triamcinolone acetonide	351 (19.0)	402 (17.7)	434 (16.6)
Oral glucocorticoids	248 (13.4)	295 (13.0)	348 (13.3)
Glucocorticoid injections	183 (9.9)	202 (8.9)	223 (8.5)
Orbital radiotherapy	53 (3.0)	62 (2.9)	62 (2.5)
Orbital decompression	21 (1.1)	22 (1.0)	24 (.9)
Strabismus surgery	15 (.8)	16 (.7)	16 (.6)
Ocular muscle transposition	1 (.1)	2 (.1)	2 (.1)
Lagophthalmos correction	2 (.1)	4 (.2)	4 (.2)

Abbreviations: TED, thyroid eye disease.

#### Comorbidities

At the time of initiation of treatment for TED and across the 3 definitions, the most common comorbidity was GD (70.8%, 71.8%, and 65.6%, respectively) followed by hyperthyroidism (37.0%, 36.4%, and 35.3%, respectively). Other comorbidities such as chronic thyroiditis and hypothyroidism were less commonly observed (Table 6).

#### Type of treatment for TED

Little to no treatment variation was found across the 3 patient definitions used. Thyroid/antithyroid preparations were prescribed for about 2 out of 3 patients (61% to 66%). Other treatments commonly prescribed include eye preparations, lubricants, and oral or injectable analgesics; anti-inflammatories; and triamcinolone acetonide (Table 6). Among eye preparations, anti-infectives, glucocorticoids, and other eye preparations were most commonly prescribed, with about 20%, 18%, and 32% prescribed each treatment, respectively. Approximately, 16% to 17% underwent treatment with oral or IV glucocorticoids. Among patients who received treatment with IV glucocorticoids (9-10%), most (80-82%) received oral glucocorticoid treatment.

Across the 3 definitions, orbital radiotherapy represented less than 3% of ophthalmological procedures followed by orbital decompression surgery. Strabismus surgery, ocular muscle transposition, and lagophthalmos correction were least common, with fewer than 1% undergoing those procedures (Table 6).

## Discussion

While previous studies considered the prevalence and incidence of GD and/or the prevalence and incidence of orbitopathy, ocular protrusion, and/or other relevant symptoms among GD patients in Japan, very few studies directly estimated the incidence and prevalence of TED [5, 16, 17, 20-25]. In this study, based on robust national insurance claims data, we established an estimate of the incidence and national prevalence rates of TED among adults for Japan. Moreover, we considered a range of definitions for TED from a strict definition based on the use of the disease name “thyroid eye disease” to file the claim and 2 broader definitions based on the use of other disease names or a combination of disease names and symptoms that are associated with TED. Despite the various definitions used to define TED patients (strict, expanded, and broad), only a modest difference in the estimated incidence and prevalence rates of TED were observed, and there were very few differences in patient and treatment characteristics by patient definition.

While previous studies that considered the age, sex, severity, and other characteristics of TED and/or GD patients in Japan have typically been limited to a single treatment center, the present study was based on a large nationwide Japanese insurance claims database [16, 17, 20-23]. Nonetheless, findings for this study concerning the age, sex, and severity of TED patients in Japan are generally consistent with previous studies conducted for Japan. Previous studies have found that a larger proportion of patients with TED are aged 35 to 59 and/or suggest a mean age in the late 40s (eg, 47.9 years old) [1, 3, 6, 15-17, 20-23]. The current research findings support previous findings and show a peak incidence at or around the age of 45 years. Previous studies have also found that a higher proportion of patients with TED in Japan are female, which was supported by our findings [16, 17, 20, 23]. While the overall incidence reported in our study was higher than those reported in Europe (4.2 per 100 000 persons in the Swedish or 5 per 100 000 persons in the Danish population), the incidence rates by sex are comparable to those reported in other countries, ranging from as low as 1.9 per 100 000 py in men to as high as 16 per 100 000 py in women [1, 4, 6, 26].

The prevalence rates reported around the world are higher than the rates reported in our study [3]. One explanation for this discrepancy might be the fact that prevalence rates around the world have mostly been estimated from GD or were based on the prevalence from a single area (county) [1, 3]. Our findings, however, are based on nationwide insurance claims databases.

The findings concerning the severity of TED in Japan are also consistent with previous studies that suggest that relatively few patients with TED (or presumed TED patients) suffer from severe disease, and, in concordance with the literature, our study reported that the majority of TED patients in Japan suffered from GD [1, 2, 17, 25, 27-29]. The present study found that the majority of patients have mild severity and/or their disease is inactive, suggesting that some patients may undergo active treatment for TED for only a short period of time.

For the current study, similar to some previous studies, the most common comorbidities associated with TED were GD and hyperthyroidism [26, 30]. While those conditions are commonly associated with TED, findings from the current study suggest that not all TED patients suffer from GD or hyperthyroidism. Although limited findings are available from previous Japanese studies on the prevalence of relevant comorbidities among TED, previous studies suggest that about 74% of adult patients with TED in Japan suffer from proptosis, and 57% and 47% suffer from lid retraction and lid swelling, respectively [25, 31]. Ocular symptoms could not be accurately assessed for the current study, as very few patients reported symptoms such as diplopia, and the reporting of symptoms appears limited in claims data.

While approximately 16% of adults overall in Japan are suspected to suffer from dyslipidemia, the current study showed that 19% to 21% of adult TED patients suffer from dyslipidemia [32]. Previous studies suggest an association between thyroid eye disease and dyslipidemia [33, 34]. Similarly, while about 12% of adults overall in Japan are suspected to suffer from diabetes, the current study showed that 14% to 17% of adult TED patients suffer from diabetes [32]. Previous studies have also suggested that diabetes is more common among patients with TED [35, 36].

Previous studies have suggested a higher proportion of patients undergoing oral or IV glucocorticoid therapy, orbital radiotherapy, and/or eye surgery; whereas for the current study a relatively small proportion of patients underwent treatment with glucocorticoids and fewer experienced orbital radiotherapy or orbital decompression surgery [5, 37]. This may relate to the small proportion of patients with an active form of the disease.

Cigarette smoking (or other types of tobacco smoking) is considered a strong modifiable risk factor associated with TED [1]. While smoking behavior is not directly ascertainable from claims data in Japan, the national prevalence of tobacco smoking in Japan is assessed each year with the National Health and Nutrition Survey conducted by the Ministry of Health, Labor, and Welfare. Results from that survey suggest that the prevalence of smoking in Japan has declined over the past several decades, with the most recent survey conducted in 2020 suggesting that about 27.1% of men, 7.6% women, and 16.7% of persons overall smoke in Japan [38]. While the overall prevalence of smoking in Japan was somewhat lower than the global adult smoking prevalence (32.6%) reported for the same year, the rate is similar to that reported in many developed countries for which many reported smoking rates are below 20% [39]. As such, the lower prevalence of TED in Japan as shown by the present study may not be related to smoking rates relative to other countries.

There are several limitations in this study. The estimates for the study are based primarily on insurance claims data for Japan, which involve some inherent limitations. Laurent et al (2023) discuss the challenges associated with the use of the insurance claims databases in Japan, including the 2 databases used for this study [40]. While the claims data are robust and come from a uniform national insurance system, both databases used leverage a subset of patients’ claims information for Japan. This might introduce bias in the results describing disease and treatment characteristics associated with age and type of facilities included. Moreover, while the findings provide one of the most robust estimates of the incidence, prevalence, and characteristics of patients with TED in Japan, the baseline data included a relatively small proportion of those aged 60 or older. Consequently, estimates of prevalence were adjusted based on the age distribution from a large hospital claims database and national population statistics. Although the JMDC database and the MDV database may not be comparable in terms of the characteristics of TED patients (eg, severity, treatment, comorbidities, etc.), the MDV database is a very large, nationwide database that is more representative of the age distribution and therefore the age distribution is not skewed. The usage of the MDV age distribution of TED patients from that database is likely to have improved the estimate of TED prevalence. Of note, while the age distribution observed for the MDV database was used for the adjustment of the prevalence estimates obtained from the JMDC database, similar adjustments were not possible for the incidence estimates and patient and treatment characteristics. As such, those estimates are likely affected by bias to the extent that fewer patients aged 60 or older are included.

Second, while the use of an insurance claims database allowed for observation of patients regardless of which facility they were treated at, if a patient withdraws from an insurance association due to a job change, etc., their treatment history after withdrawing cannot be confirmed. A wash-out period was used for the analysis of incidence and patient and treatment characteristics, in order to avoid inclusion of persons for which only partial data were available. However, this process may also have excluded certain patients. Third, our findings may only provide an approximation of severity and disease activity for TED in Japan, because the definitions of severity and disease activity supported by the treatment guidelines, which include a consideration of instruments such as CAS score, which is not captured in claims databases, could not be used. Instead, definitions of severity and disease activity based on the patients’ treatment were used. Lastly, claims databases in Japan may not capture the use of off-label treatment. For example, immunosuppressants are mentioned as a treatment modality for TED, but they are not indicated for TED in Japan, so their usage may not be captured by claims data. However, a recent survey conducted among American and European Thyroid Association members found that immunosuppressants are used by very few patients, and a similar trend is likely for Japan [2, 41].

In conclusion, while the incidence and clinical characteristics observed for TED in Japan were consistent with what was reported in previous studies, the prevalence observed for this study was lower than the prevalence reported by other studies across different geographic locations.

## Data Availability

Original data generated and analyzed during this study are included in this published article or in the data repositories listed in References.
